# Young adults’ use of food as a self-therapeutic intervention

**DOI:** 10.3402/qhw.v9.23000

**Published:** 2014-04-15

**Authors:** Elisabeth Von Essen, Fredrika Mårtensson

**Affiliations:** Department of Work Science, Business Economics and Environmental Psychology, Swedish University of Agricultural Sciences, Alnarp, Sweden

**Keywords:** Emotions, lived body experience, nature, organic food, positive psychology, resilience, restoration, stress, vegetarianism

## Abstract

The aim of this study was to investigate how young adults use their lived body as a starting point for lifestyle explorations and as a strategy for well-being. The transcripts of 10 interviews with persons 18 to 33 years old, collected in Sweden, were analysed for variation in the practises and experiences related to this way of using food. An application of the descriptive phenomenological psychological research method guided the process. The young adults were: (1) listening to the body; (2) moderating conditions and feelings; (3) developing vitality and resilience; (4) creating mindful space for rest, and (5) participating in creative activity. The results show how young adults perceive their choice of food and related practises associated with positive feelings and experiences as ways to promote well-being and mitigate different problems in life. The usefulness of knowledge about how young adults try to use food for self-therapy by enhancing mind-body awareness is discussed in relation to health issues and food-related interventions.

Many public health problems of our time are related to food, such as overweight, diabetes (SNIPH, 2009), and eating disorders, as well as chronic inflammation and depression (Lucas et al., 2014). Among young adults, unhealthy lifestyle choices with a lot of junk food are easily available and different symptoms of anxiety are common (Skrove, Romundstad, & Indredavik, 2013). As good eating habits play an important role in health and well-being (McDade et al., 2011; WHO, 2010), it becomes important to learn about these as part of contemporary lifestyles and everyday practises among young adults (Bukhari, Fredericks, & Wylie-Rosett, 2011).

This study takes its departure in the work of the existential phenomenological philosopher Maurice Merleau-Ponty (1945/2012), describing how we use our body to explore the world and to express and reveal ourselves as active and intentional subjects. Our bodies continuously contribute to our experiences (Gallagher, 2005; Gallagher & Zahavi, 2012). When directing attention to specific regions of the body, we become conscious of subtle, inner sensations (Kabat-Zinn, 2013; Pagis, 2009) in a way that facilitates reflection on our present physical and mental state (Gallagher & Zahavi, 2012; Stern, 2004). If we are capable of considering how food contributes to our being vital and happy or depressed, it can become part of a strategy that “shapes the way we perceive the world” (Gallagher & Zahavi, 2012, p. 156) and a tool for change (Damasio, 1999).

Food intake is associated with many different enjoyable everyday activities, which enable us to show care and togetherness (Daniels, Glorieux, Minnen, & Van Tienoven, 2012) and lead a good life (Macht, Meininger, & Roth, 2005). Not infrequently, the preparation of specific food and procedures of eating are associated with positive emotions (Kaufmann, 2010; Macht et al., 2005) important for a person's development, identity formation (Bisogni, Connors, Devine, & Sobal, 2002; Fischler, 1988), and self-determination (Pelletier, Dion, Slovinec-D'Angelo, & Reid, 2004).

The stress put on making conscious food choices is common for many popular diets supposed to be part of a strategy for health (Baer, Fischer, & Huss, 2005; Harrison & Jackson, 2009; Williams, Veitch, & Ball, 2011). Mindful eating (Bays, 2009) implies staying aware of physical and emotional sensations while eating (Moor, Scott, & McIntosh, 2013). The literature describes how mindfulness, as a form of non-judgmental body awareness (Kabat-Zinn, 2013; Mehling et al., 2011) increasing curiosity and openness towards body sensations (Bishop et al., 2004), can counteract “cognitive dominance” and help the mind and heart become an integrated whole (Khong, 2011). Studies show how practises of mindfulness can be related to an increase in well-being and self-compassion (Baer, Lykins, & Peters, 2012; Neff & McGehee, 2010), success in diet change (Dutton, 2008) and weight reduction (Gilbert & Waltz, 2010), insulin resistance in diabetes (Sahay, 2007), and less everyday stress (Köhn, Lundholm, Bryngelsson, Anderzén-Carlsson, & Westerdahl, 2013).

Well-being is a multidimensional concept object of many health-studies today (Dodge, Daly, Huyton, & Sanders, 2012; Snyder & Lopez, 2007). In positive psychology, the concept refers to all those positive feelings that are targeted towards one's own person, and aspects of life characterized by optimal functioning and flourishing (Fredrickson & Losada, 2005). Of importance is the opportunity for explorative behaviour (Banfield & Burgess, 2013; Wilkinson & Chilton, 2013) and creative activity (Heenan, 2006), characterized by a balance between resources and demands (Dodge et al., 2012). Studies show how positive feelings and a broadening of a person's perspective can make it easier to manage setbacks and build resilience (Fredrickson, 1998, 2001). More specifically, studies show that positive experiences of nature can contribute to recuperation and personal development (Adevi & Mårtensson, 2013; White, Pahl, Ashbullby, Herbert, & Depledge, 2013) and how a positive attitude to organic products is often related to such experiences (Hjelmar, 2011).

An earlier study of organic food choices among young adults showed that food can be part of an overall life strategy for health and well-being, as described in the following general structure of this phenomenon (von Essen & Englander, 2013):

The lived psychological meaning of choosing a healthy lifestyle based on an organic diet is experienced by the young adult as an overall complexity in terms of discovering a sense of independence and self-regulation. The lived body is seen as the starting point and the vehicle of a special skill, in which life is explored through nutrients, flavours, and food texture. The exploration through this special skill creates an enlightened life strategy in terms of well-being and vitality. The perceived healthy lifestyle is constituted by a set of values and ethical standards that transcends the individual and includes the well-being and vitality of the next generation, including animals and nature. A narrative self is disclosed within the context of the choice of healthy lifestyle that reflectively relates to, emerges from, and persists in one's emotional and relational food memories. (p. 4)

The aim of this study was to investigate two specific aspects (or constituents) of this overall phenomenon, namely how young adults use their lived bodily experiences of food as the starting point for lifestyle exploration and how they use these experiences as a life strategy for well-being and vitality.

## Method

### Sample and procedure

A sample of ten^2^ interviews evaluated as information rich were selected from a sample of 30 interviews which were part of a larger research project in which persons showing interest in organic food through social media^3^ were contacted (von Essen & Englander, 2013). The first author carried out the interviews face-to-face in the participants’ homes. The interviews were semi-structured and lasted between one and a half and two hours. The participants were asked to talk in detail about everyday experiences related to their choice of organic food and strategy for a healthy lifestyle. The interviews were transcribed word for word. The strategy was time-consuming with its focus on detail and closeness to data (Englander, 2012), but provided the depth needed to study lived experiences (Giorgi, 2009).

### Data compilation and analysis

The general structure of the phenomenon under study—young adults^1^ choosing a healthy lifestyle based upon an organic diet—is based on transcripts from three individuals, according to the recommendations of the descriptive phenomenological psychological method (Giorgi, 2009). This phenomenon contained in it four different aspects, so-called constituents: (1) The lived body as the starting point for lifestyle exploration; (2) a narrative self through emotional-relational-food-memories; (3) a life strategy for well-being and vitality; and (4) a personal set of values in relation to ethical standards (von Essen & Englander, 2013). In this study, we investigated variations in the lived experience across the larger sample of ten individuals in two of these constituents: that is, “the lived body as the starting point for life exploration” and “a conscious life strategy for well-being and vitality.” It was considered possible to remain within the realm of the in-depth research strategy of the method selecting two, out of the four constituents (Giorgi, 2009).

The overall strategy for data analysis was made according to three steps in the descriptive phenomenological psychological method (Giorgi, 2009): (1) the interviews were read through in order to obtain a comprehensive view of the interview material; (2) units of meaning were defined and delimited; and (3) data from the interviews was translated to a more phenomenological, psychological level in a third-person perspective. The latter means a focus on how the participant lived in the situation and a transformation of each unit into the language of psychological science (Giorgi, 1979, 2009).

The fourth step of the method in which one uncovers a general structure of the phenomenon has already been carried out in an earlier study (von Essen & Englander, 2013), and the aim of this study was to explore variations of this phenomenon across a larger set of data. This is in line with a suggestion by Amedeo Giorgi (1979) that such a strategy could add useful psychological knowledge. One could liken the procedure with the growth of a tree, where the general structure of the phenomena is the stem, the four constituents are the branches, and the five different types of practises and experiences presented in this paper are the leaves of the tree. The specific strategy (Giorgi, 1979) was to look across data for variation in the appearance of the phenomenon unfolding in relation to the psychological meaning implied by the two selected constituents. It was a search for clusters of experiences related to specific life conditions or forms of action. Underpinning this perspective is the assumption that all subjects had the same general cause of action, even if some emphasize the role of the body, others the perceptual process, and still others talk about motivation. In this way, we ended up with five different types of practises presented in the results section with citations enriching each type, further emphasizing the empirical variation of the phenomenon (Giorgi, 2009).

The ambition throughout all the steps was the scientific phenomenological reduction, which means reducing the participants’ experience to a phenomenon, treating it as real experiences even though it does not “exist exactly the way it is experienced” (Giorgi, 2009, p. 90). One tries to mentally bracket prior knowledge about the phenomenon and any theory, and emphasize how the experience presents itself to the describer. The ambition with this strategy of free imaginative variation, is to clarify various psychological aspects of the experience without adding to or subtracting anything from a person's statement, and to clarify the descriptions until one can no longer vary the descriptions without changing the concrete experiences (Giorgi, 2009). The focus on detail and closeness to data throughout the process provide the complexity and depth needed to study lived experiences (Englander, 2012).

### Ethical considerations

This study was conducted according to the ethical guidelines published by the Swedish Research Council (Gustafsson, Hermerén, & Petterson, 2011). Information was provided to the interviewees about consent, confidentiality, and how the data would be used, both during recruitment and at the interview. It was made clear that it was optional to respond to specific questions and to disrupt and withdraw from the study at any time. Several interviewees recalled periods of stress and vulnerability in life and touched on personal information that in some respect demanded special attention. Care was taken to close the interview in a good way. The material has been decoded, treated confidentially, and only made accessible to the research group. The interviewer has a master's in psychology, and is a trained psychotherapist.

## Results

We know that young adults with a preference for organic food actively use their bodies and their senses to explore what possibilities there are to make food part of a strategy for health and well-being (von Essen & Englander, 2013). This study investigates the variation on this theme, with the experiences and practises making up the lifeworld of the young adults grouped into five different types of approaches, describing how they are: (1) listening to the body; (2) moderating conditions and feelings; (3) developing vitality and resilience; (4) creating a mindful space for rest, and (5) participating in creative activity. A commonality among these experiences is their ambition to be mindful about their choices and activities related to food. After this elaboration of the experiential and psychological meaning of various experiences, we will reflect on the practical implications for health and well-being when young adults get the ambition to turn food choice and food preparation into mindfulness practise.

### Listening to the body

The young adults reflected on the intelligence of the body giving them the ability to recognize positive and negative body states and feelings related to food. When directing their attention inwards, “listening to the body” and to their “gut feelings” (P24), they experienced themselves being aware of variations in emotional tone. They were updated and guided in their choice of food and mindful of when there was a need for change or whether they craved a specific ingredient, liquid, or nutrition, across situations and predicaments.

One woman (P24) perceived an increased reliance on her body intelligence after having gone through periods of stress and mental exhaustion when she had lacked any sense of taste and had no sense of being full, or other bodily feelings. She remembered how she then had to rely on her cognitive ability and “think out” what was good for her. Her experience now was that she would have to reconcile herself to having these strong feelings and accept new things about herself, or to not having any feelings at all:

I've discovered now that I have strong feelings and I know how it feels when I can't feel anything at all … Now I'm working actively with tasting. If I don't listen to my body, everything ends up here in my head.

One man (P2) recognized a particular sensitivity to food during periods of illness and mental imbalance when feeling vulnerable and exposed and his body was depleted from feelings of hunger. He then felt more considerate about choosing food that was easy for the body to digest and process such as salad, sprouts, wheatgrass drinks, or a piece of fruit such as grapefruit that is “fresh and clean,” and not any food that “takes energy”:

The last thing I can think of is eating very spicy food, certain consistencies or certain smells and impressions, which I don't really appreciate, like garlic.

One woman (P26) experienced how the body craved specific ingredients when she had been eating too one-sidedly and after intense physical activity, but also how she had to learn to distinguish between feeling a lack of vital substances, such as the vitamins in vegetables, and the craving of sugar as a bad habit or addiction.

### Moderating conditions and feelings

Food was also used to counteract negative feelings and conditions. Positive feelings related to food were experienced as helping to create a more dynamic and flexible situation in which one stayed more attentive to variations and nuances in emotion as a base for self-regulation.

One man (P2) explicitly perceived how he used food to moderate feelings in order to counteract depression or get rid of anger or annoyance and instead gain strength and energy. He experienced how he could solve problems by “eating better,” and handle situations in life by preparing specific food in specific ways, in a similar way as his mother had done during childhood:

I bake buns, preferably a double portion so that I can really work hard with the dough. It's a good way to get rid of anger.

One woman (P1) experienced how she stressed the close connection between how she felt and how and what she ate, as being to some extent based on a body complex from childhood. Her elaborated everyday routines related to food helped her to stay emotionally neutral and at peace also when under pressure. Standardized routines and rules for food were experienced as helping her stay stable and well-functioning, also when she otherwise felt threatened to become overwhelmed by feelings. She felt how during free time she tried to balance her strict schedule by rewarding herself with a more relational event and being more accepting of herself and her immediate preferences:

I look forward to what I am going to eat at the weekend when I am allowed to eat dinner with someone, and I know that we are going to make good food and there'll be a dessert.

She perceived a fine balance between the stability gained by adhering to this schedule and the possible disruption if she did not, for example, if someone gave a dinner in the middle of the week:

I feel that I can be flexible but that it's always a fine balance. Because if I happen to eat something that I didn't think I should have eaten, then the result can be that I don't feel so good later.

### Developing vitality and resilience

The participants described how they had become aware of using food to maintain health and strength, as a way to gain resistance to different kind of stressors and pressures and to reach equilibrium.

One man (P23) felt how the right food made his body more strong, reliable, and vital and himself more capable and able to carry through everything that he wanted and needed to do in his daily life, with fewer mood swings as a result. Being a vegetarian, he was attentive to food containing “some kind of protein.” He described the obstacles to health and vitality when obliged to eat at restaurants lacking nutritious vegetarian food or when he was only able to afford wokked vegetables and rice which only keeps him full for a short while.

One woman (P1) experienced that she could be “very extreme” about food being nutritious and healthy and related being stable, feeling well and full of energy with eating regularly every third hour:

It's important for me that my body is well from the inside and I rely on my blood sugar being even, so that I don't feel unwell.

One woman (P24) reflected on how the choice of “real food” that was organic and vegetarian had become part of an active strategy to recover vitality and gain psychological and physical strength. From being a stress factor in her life, she experienced how food had become a way to be respectful and considerate about herself and to gain self-confidence, even if she still felt bad and depressed some days. She had started to reflect on what she had exposed her body to earlier in life and wanted to “get out what felt stiff.” She tried to develop a new way of living by learning from “small things in life” and by avoiding eating badly, like having sandwiches for dinner or a snack.

Another woman (P30) perceived food choices as part of an overarching strategy to not lose sight of the most important things, to counteract worries about the future, and to make good choices in life, such as devoting herself to school. She felt that eating specific food had become a way for her to look after herself and stay mentally well, strong and capable:

I think instead about how much I appreciate organic food and I notice that I feel well from having a properly cooked meal with healthy raw ingredients which make me and my body livelier and much happier.

### Creating a mindful space for rest

The preparation and the eating of food at a slow pace without any disturbances, is another aspect of the participants being aware of their relation to food. They describe it as a way to feel inner calmness, relax and recuperate and how “everything fell into place” (P24) with this new approach to food.

One woman (P13) pondered how simple and repetitive activities, such as baking and chopping vegetables, had become opportunities to slow down, relax, and separate herself from disturbing thoughts and everyday demands. These activities allowed her body and brain to switch off into meditative detachment and experience how time could pass without her noticing it. She compared this with experiences made during yoga, mindfulness, and canoeing:

By being totally focused on what I do and disappearing into an activity for a while, my brain switches off and time passes without me noticing. It's similar to other things like for example when I swim or canoe. It becomes a form of relaxation, which means I can switch off all the other thoughts or things I have around me.

Another woman (P24) also related food activities with other activities considered as characterized by mindfulness, all giving her a chance to “live for the moment.” She felt that it was important for her to take it easy while eating, to relax and concentrate, and not read while eating. She described how food helped her to slow down, lead a more calm life, pay attention to what was going on in her body and how the body turned more supple and calm:

Food needs to give me inner peace, in my body, like being outdoors, and then it gets calm.

### Participating in creative activity

Cooking food was also described as a creative activity, which the participants associated with personal development, self-realization and social togetherness. They described how they preferred to compose their own dishes and make experiments, rather than follow recipes or eat ready-made food. It was an activity experienced as demanding their full moment-to-moment attention, empathy, imagination and inventiveness.

One man (P23) reflected on the importance of composing food from scratch and the challenge of finding good ingredients and combining these in his own ways:

It's not cooking to put a ready-made factory sausage into the oven … that's warming up and I really want to compose my food myself and experiment to see what works for me.

A woman (P25) described the joy and mental space involved in the handiwork of food making. She described how vegetarian dishes containing many different ingredients demanded a lot of cutting that gave her the opportunity to smell, taste and experiment.

Another woman (P13) experienced how food preparation at the weekend could grow into a long step-wise process when choosing dishes demanding hands-on creativity and close attention to detail, such as when making sauces and fillings or a dough rise from yeast or folding rice paper. She described how the choice of food that is “a bit fiddly” made it possible to transfer her time and energy from various demanding tasks and worries to the joys of food preparation.

One man (P2) reflected on homemade bread as food with more flavour and “more alive with things happening to it.” He also stressed the role of key people in life being present for the motivation to prepare food and the importance of company when cooking and eating. Preparing food without the possibility to share with others bored him:

I can't be bothered to make an effort and do that last little bit.

## Discussion

The young adults with a preference for organic food describe how they think that mindful practises and experiences related to food have helped them to transcend to more enduring and positive emotional states, increasing their vitality (Stern, 2010), resilience (Fredrickson & Losada, 2005), self-compassion (Baer et al., 2012), and “sense of agency” (Gallagher & Zahavi, 2012). They expect their mindful choices and mindful practises related to food to make it easier for them to manage pressures, set boundaries, and develop their capacity for positive feelings of happiness, curiosity, and love (Fredrickson, 1998). They talk about a lifeworld with many direct and indirect references to “mindfulness” (Bishop et al., 2004; Kabat-Zinn, 2013; Mehling et al., 2011). Food is described as a tool through which they experience and make the world part of their body in a way that enhances their mind-body awareness.

They describe the use of organic food—usually also implying vegetarian—to a positive development of many different psychological and physiological factors (Williams et al., 2011). Similar to studies on vegetarian food choice, participants expect organic food to prevent lifestyle-related illnesses such as diabetes, heart- and circulatory diseases, cancer, and obesity (McDade et al., 2011), but also to protect them from poor mental health such as anxiety and depression (Skrove et al., 2013). They also describe how food helps them to open up to new information and experiences (Fredrickson, 1998) in a way that broadens their thought-action repertoire (Fredrickson, 2001). It is supposed to increase their capacity for bodily-based practises and make them less reliant on cognitive information and skills (Bukhari et al., 2011). In general, the young adults seem to have the ambition to become more reliant on everyday activities to which they can “tune in” (Kabat-Zinn, 2013) and experiment with how they relate to the physical surroundings.

When feeling especially vulnerable, they seem to “take a step backward” and rely on a “bodily intelligence” with moment-to-moment awareness and self-observation (Mehling et al., 2011), to better understand their own expressions of weakness, vitality, and balance (Gallagher & Zahavi, 2012). Just like Kabat-Zinn (2012), they describe how different senses mediate various experiences, which continually update them about the world and create meaning. Sometimes they even try to prevent negative emotions expected to be approaching (e.g., related to an abandonment) by getting involved in some cherished food practise. Intimate and bodily-based experiences of food appear to broaden their sphere of lived reality with the smells and tastes of specific dishes and ingredients also related to things such as season and social context. In the literature it is described as an ability to stay present in the moment and vigilantly observe the body's reactions (Stern, 2004), in order to manage negative feelings and create a protective shield against external pressures (Khong, 2011).

Their descriptions related to food have many similarities with experiences described by people taking part in mindfulness training (Kabat-Zinn, 2013) meditation (Pagis, 2009) and garden therapy (Adevi & Mårtensson, 2013). There is a stress on simultaneous experimental and existential interaction with the surroundings, relying on pre-reflective modes of being, (Banfield & Burgess, 2013; Wilkinson & Chilton, 2013) and the opportunity for restorative experiences (Kaplan, 1995). Examples of this are when they describe how food stops time and takes attention away from everyday worries (Gallagher & Lopez, 2007), the relaxation of repetitive movements during preparation (White et al., 2013), and how food helps to create an inner space for rest, meditative calmness, and empathy for themselves (Baer et al., 2012; Neff & McGehee, 2010).

As much as a food practise can be an inward looking activity, it could be argued that food is an outward bound, social, and creative activity (Heenan, 2006). It becomes part of a strategy trying to make the surrounding world more understandable and friendly, possible to approach and challenge (Dodge et al., 2012), and relate to in new ways (Pagis, 2009). As in earlier studies (Macht et al., 2005), meals alone are generally described as less positive than the ones with family or friends. Daniels et al. (2012) have described how the arrangements of meals in various family constellations are related to the management of emotions. Food preparation in itself is likely to become a way to show love and care, finalized by the intimate act of sharing the food which in some way has become a part of themselves during the process (Gallagher & Zahavi, 2012). Participants of garden therapy even associated the act of eating home-grown vegetables with the holy communion (Adevi & Mårtensson, 2013).

### Method and limitations

The phenomenological perspective seems very applicable in the study of new bodily oriented diets and the life practises of many young adults. A limitation however, could be that all the participants were recruited from urban areas in which the options to choose organic food are high. On the other hand, young people tend to move to cities and are attracted to new trends regardless of where they live. It can also be experienced as a flaw that the analysis is based on a larger number of interviews than the descriptive psychological phenomenological method originally was designed for. This means that some parts of each interview were excluded from the later steps of the analysis to make the material manageable. We tried to counteract the risks of fragmentation by reading through the material several times to detect idiosyncrasies. On the other hand, our choice of method resulted in a richer and thicker description of lived reality, possibly making it easier to discuss this life style strategy in its everyday context and across settings.

### Conclusions, implications, and suggestions for future research

The results show how food becomes part of a strategy for health among young adults including five different types of practises related to food in which they mindfully work with the mind–body connection to find an inner room for rest and creativity and become more open, compassionate, and curious. They seem to regard food as an extension to the body that they can use for existential investigations when trying to counteract stress and overcome feelings of powerlessness. It is a description of how they try to make food part of a self-therapeutic intervention with different mindfulness-oriented practises. In some respects, this mindful eating and preparation of food could be regarded as an extreme form of nature-based therapy in which we not only choose a specific garden room for restoration (Adevi & Mårtensson, 2013), but make the nature we prefer part of our body. The incorporation of the world into the mouth when eating is, in the sense of Merleau-Ponty (1945/2012), an intimate contact with the surrounding world indeed.

The insight gained from this study could possibly be useful in the counselling and development of interventions related to mental health and food disorders, by focusing attention on the potentially very dynamic relation between body and mind, for example, when a person is obliged to cope with a new diet. However, one need to be attentive to the fact that this very dynamic relation to food could also be part of a problematic strategy in which trying to eat healthy food takes up too much of a person's life. The eating disorder, orthorexia nervosa (Zamora, Bonaechea, Sánchez, & Rial, 2005), related to eating pure or organic food (Bratman & Knight, 2004), also implies a controlling and restrictive approach to food which has some similarity with the “health strategy” described in this article. We suggest that the dynamic relation to food as a health strategy and part of different mindfulness-oriented practises be investigated critically in future studies.

## Notes

1 Following Young et al. (2011) in this context, young adult refers to the age span between 18 and 35.

2 P1 is a 25-year-old woman, studying and living in a small town in southern Sweden. P2 is a 30-year-old man, studying and living in a large city in southern Sweden. P13 is a 33-year-old woman, employed and living in a big city in central Sweden. P16 is a 24-year-old woman on maternity leave with a small child, living in a small town in central Sweden. P17 is a 30-year-old woman, studying, living in a medium-sized city in southern Sweden; P23 is a 28-year-old man, employed, living in a large city in southern Sweden. P24 is a 30-year-old woman, employed, living in a large city in central Sweden. P25 is a 25-year-old woman, studying and working part-time, living in a mediumsized city in southern Sweden. P26 is an 18-year-old woman, studying, living in a small town in southern Sweden. P30 is an 18-year-old woman, studying, living in the country in central Sweden.

3 The 30 participants were recruited through requests for participation through various social media: Facebook, blogs, and digital newsletters with a national distribution and a focus on organic food. Social media is the information channel that young adults use today to obtain instant knowledge. The inclusion criteria for participating in the study were being between 18 and 35 years old and having an interest in organic food. All who signed up and met the criteria were interviewed.
